# Risk factors for sexual dysfunction in Romanian women with type 1 diabetes mellitus and chronic autoimmune thyroiditis: a comparative cross-sectional study

**DOI:** 10.1186/s13098-020-00602-7

**Published:** 2020-10-27

**Authors:** Adriana Gherbon, Mirela Frandes, Deiana Roman, Diana Anastasiu-Popov, Romulus Timar

**Affiliations:** 1 Second Department of Internal Medicine - Diabetes, Nutrition and Metabolic Diseases, “Victor Babes” University of Medicine and Pharmacy, Timisoara, Romania; 2 Clinic of Diabetes, Nutrition and Metabolic Diseases, “Pius Brinzeu” Emergency Hospital, Timisoara, Romania; 3 Department of Functional Sciences - Medical Informatics and Biostatistics, “Victor Babes” University of Medicine and Pharmacy, 2 Eftimie Murgu, 300041 Timisoara, Romania; 4 Department of Obstetrics-Gynecology, “Victor Babes” University of Medicine and Pharmacy, Timisoara, Romania

**Keywords:** Female sexual dysfunction, Type 1 diabetes mellitus, Chronic autoimmune thyroiditis, Depression, Polyneuropathy, Insulin pump

## Abstract

**Background:**

Female sexual dysfunction (FSD) is one of the chronic complications of diabetes as is male sexual dysfunction, but the former is less studied. Therefore, the aim of this study was to assess of the prevalence and risk factors associated with FSD in Romanian women with type 1 diabetes mellitus (T1DM) and chronic autoimmune thyroiditis (CAT).

**Methods:**

The study sample included 104 Romanian women with both T1DM and CAT, and 101 Romanian matched controls with only T1DM. The presence of FSD was established using two validated tests: The Female Sexual Function Index and the Female Sexual Distress Scale-revised. The presence of depression was assessed using Beck’s Depression Inventory-II.

**Results:**

We found that almost half of the women in the group with T1DM and CAT presented with sexual dysfunction (49 vs. 33.7% in the control group; p = 0.025): 27.9 vs. 8.9 (p < 0.001)—desire, 23.1 vs. 7.9% (p = 0.003)—orgasm, 21.2 vs. 5.9% (p = 0.002)—lubrication, 17.3 vs. 6.9% (p = 0.023)—arousal, 9.6 vs. 1% (p = 0.006)—pain, and 20.2 vs. 9.9% (p = 0.040)—satisfaction problems. Multivariate regression analysis revealed that age was a significant risk factor for FSD, along with DM and body mass index. Coexisting CAT, polyneuropathy, depression, and the use of insulin pumps were significant risk factors for FSD.

**Conclusions:**

Women with T1DM and CAT are more likely to present with FSD than those without. It is important for patients to address depression, if present, and exercise caution while using insulin pumps. Moreover, DM complications such as polyneuropathy are significant risk factors for FSD; thus, it is crucial to ensure satisfactory glycemic control and optimal DM management.

## Background

Female sexual dysfunction (FSD) is one of the chronic complications of diabetes, as is male sexual dysfunction, but the former is less frequently studied. Sexual dysfunction refers to difficulty in performing normal sexual activities and experiencing physical pleasure, desire, arousal, or an orgasm. According to the Diagnostic and Statistical Manual of Mental Disorders (DSM-5), sexual dysfunction refers to extreme distress during normal sexual activity for a minimum of 6 months in the absence of any substance or medication-induced sexual dysfunction [1]. FSD includes dysregulation of desire, arousal, lubrication, orgasm, and satisfaction and pain sensation [2]. This complication affects the quality of life of patients with diabetes [3]. The main factors involved in the occurrence of FSD are related to reproduction, relationships, sociocultural specifics, and health problems such as chronic medical conditions and related treatments [4].

Type 1 diabetes mellitus (T1DM) is an autoimmune multifactorial disease caused by genetic susceptibility and environmental triggers such as viral infections, toxins, or dietary factors [5]. T1DM usually occurs in children or young adults (occasionally in older adults) [6], and it is associated with other autoimmune diseases, the most common being chronic autoimmune thyroiditis (CAT) (incidence rate of 15–30%) [7, 8]. This disease is characterized by hypothyroidism and requires hormonal treatment. These hormones induce hyperglycemic effects and even slight changes in their level increase the risk of hyperglycemia [8]. Patients with hypothyroidism are at a high risk of developing dyslipidemia and increased intima-media thickness, vascular stiffness, endothelial dysfunction, and hypercoagulability. All these factors could contribute to the high prevalence of cardiovascular complications in diabetic patients with hypothyroidism [9].

In 2019, the estimated number of people with DM was 463 million worldwide. This number is expected to increase by over 51% over the next 20 years (700 million adults in 2045). In Europe, the number of persons with DM was 59 million in 2019, and it is expected to increase by 15% by 2045 (68 million). The number of children with T1DM worldwide is 1,110,100, with more than a quarter (27%) located in Europe [10]. In Romania, the prevalence of DM in 2017 was 10–13%. According to the PREvalence of DiAbeTes mellitus, prediabetes, overweight, Obesity, dyslipidemia, hyperuricemia and chronic kidney disease in Romania (PREDATORR) study, the prevalence of DM in the western region of Romania was 8.2% and that among women aged 20–40.8 years was 2.4% [11].

Very few studies have focused on sexual dysfunction in women with DM. Moreover, many studies have focused on FSD related to T2DM and not T1DM [12]. The studies involving men showed a correlation between sexual dysfunction and duration of diabetes, glucose control, cardiovascular and neurological complications, older age, antihypertensive treatment, comorbidities, body mass index (BMI) [13], tobacco use, and medication [14]; in women, some studies showed a correlation between FSD and duration of diabetes. A significant correlation has been found between FSD and neuropathy (both sensitive and autonomic neuropathy) [15]. The most common risk factor for sexual dysfunction in women with diabetes is depression [16]. Furthermore, some studies outlined other psychological risk factors such as problems of self-image, tiredness, and dependency on others [17, 18].

Thyroid diseases are considered risk factors for sexual dysfunction [19]. Both hypothyroidism and hyperthyroidism were found to be associated with erectile and ejaculatory dysfunctions in men [20], respectively, and with impairments in desire, arousal/lubrication, orgasm, and satisfaction and pain during intercourse in women [21, 22]. The development of sexual dysfunction is attributed to the effect of thyroid hormones on circulating sex hormone levels through peripheral and central pathways, indirectly leading to psychiatric and autonomic deregulation and causing impairments in sexual function. Correction of thyroid dysfunction to the normal status is reportedly associated with the resolution of sexual dysfunction in both men and women [23].

Studies on FSD in women with T1DM have reported incidence rates between 18 and 71%. An odds ratio of 2.27 in DM1 patients was reported in one study [24]. The Epidemiology of Interventions and Complications (EDIC) study and urologic assessment data therein provided evidence of a direct link between sexual dysfunction and autonomic neuropathy (particularly cardiovascular autonomic neuropathy) in women with T1DM [25]. However, less-evident factors, such as psychiatric problems related to a chronic, lifelong disease, can contribute to the development of FSD in DM patients as well [26].

Both T1DM and CAT are autoimmune diseases, representing the type 3 autoimmune polyglandular syndrome (APS). APS includes multiple endocrine organ defects [27]. Our sample included women with both T1DM and CAT since we wanted to investigate whether the association between these two autoimmune diseases influenced the prevalence of FSD. The purpose of this study was to assess the prevalence and risk factors associated with sexual dysfunction in Romanian women with T1DM and CAT.

## Methods

### Study design and patients

This study was a comparative cross-sectional, non-interventional study, involving 104 non-menopausal women with T1DM and CAT, aged 18–40 years with an active sexual life. They were randomly selected from the outpatient clinic at the Timisoara County Emergency Hospital from January 1, 2018 to December 31, 2018. The control group consisted of 101 Romanian patients with only T1DM.

All patients presented with T1DM and were treated with intensified insulin therapy with four daily injections or insulin pumps.

The inclusion criteria were as follows:

- Age between 18 and 40 years
- Presence of both T1DM and CAT in the study group and only T1DM in the control group
- No health problems other than complications of DM and CAT
- Active sexual life and not menopausal

The exclusion criteria were as follows:

- Absence of a sexual partner in the last 12 months
- Presence of an active/untreated psychiatric disease

We excluded menopausal women because, in this state, some sexual dysfunctions occur, such as lack of desire and libido, predominantly associated with decline in serum levels of estrogen and vaginal dryness [28]. Aging also has a strong impact on sexual function in women, specifically in the areas of desire, sexual interest, and frequency of orgasm. These changes occur because of the gradual decline in testosterone levels in most women with aging [29].

This study was conducted in accordance with the Declaration of Helsinki. All included patients provided informed consent. Our study was approved by the Institutional Ethics Committee (The Ethics Committee of the Emergency Hospital, Timisoara), Number 52083/2019. All data were anonymized before analysis.

We collected data from the patients’ medical records and three validated questionnaires on sexual dysfunction and depression, namely the Female Sexual Function Index (FSFI), Female Sexual Distress Scale-Revised (FSDS-R), and Beck’s Depression Inventory-II scale (BDI-II) as well as collected clinical data, anthropometric parameters, and results of biochemical investigations. The data collected from the medical records included general information, obstetric/gynecologic history, and anamnesis of sexual activity.

### Clinical, anthropometric, and laboratory data

We determined anthropometric parameters such as height, weight, BMI, abdominal circumference, and blood pressure and examined biochemical parameters for glycemic balance, i.e., fasting blood glucose, glycated hemoglobin, and lipid profile (total cholesterol: high-density lipoprotein [HDLc], low-density lipoprotein [LDLc], and triglyceride [TG]); for kidney function, i.e., eGFR, albuminuria, serum creatinine, and creatinine/albumin ratio; and for thyroid gland function, i.e., thyroid-stimulating hormone (TSH), thyroxine (FT_4_), and antithyroid antibodies.

The criteria for the diagnosis of T1DM were fasting plasma glucose levels > 126 mg/dL, symptoms of hyperglycemia (polyuria, polydipsia, and unexplained weight loss with random plasma glucose ≥ 200 mg/dL or 2-h plasma glucose ≥ 200 mg/dL during an oral glucose tolerance test), and glycosylated hemoglobin (HbA1c) ≥ 6.5% (≥ 48 mmol/mol). In some cases, the diagnosis was confirmed with low C-peptide levels and presence of one or more autoimmune markers of diabetes autoantibodies to glutamic acid decarboxylase, islet antigens (IA2 and IA2-beta), and the zinc transporter ZnT8 [30].

The diagnosis of subclinical autoimmune thyroiditis (Hashimoto’s thyroiditis) was based on high levels of TSH and the presence of at least one thyroid autoantibody on two or more consecutive occasions, and/or with ultrasonographic findings of thyroiditis. Clinical hypothyroidism was associated, in addition to the above, with low FT_4_ levels and/or the presence of goiter [31].

The patients were screened for micro- and macrovascular complications with the following protocol: ophthalmologic examination, podiatric examination (vibration, tactile, pain, and thermic sensation), ankle/brachial index, electrocardiography, nerve velocity conduction, tests for cardiac autonomic neuropathy, and ultrasonography of the thyroid gland.

Venous blood samples were collected from all participants on the day they were invited to participate in this study to determine the levels of HbA1c, TSH, FT_3_, FT_4_, and antithyroid antibodies and the lipid profile. The hemoglobin A1c (HbA1c) level was measured using the National Glycohemoglobin Standardization Program (NGSP)-standardized and Diabetes Control and Complications Trial (DCCT)-compliant immune turbidimetric assay (Hoffman-La Roche Ltd., Basel, Switzerland), with an inter-measurement coefficient of variation of 1.64% according to the manufacturer’s specifications. The reference range was 4.8–6.4% [30]. Lipid profile was assessed using spectrophotometry (enzymatic-colorimetric), and the following reference values were set: total cholesterol < 200 mg/dL, triglycerides < 150 mg/dL, LDLc < 100 mg/dL, and HDLc > 50 mg/dL.

Antithyroid peroxidase (anti-TPO) and antithyroglobulin (anti-Tg) antibodies were detected in venous blood samples using the electrochemiluminescence immunoassay (ECLIA). The upper normal limit for the anti-TPO antibody was set at 34 UI/mL and for the anti-Tg antibody at 115 U/mL [31]. Values greater than these cut-off values indicated positive results. For the evaluation of thyroid function, TSH, FT_4_, and FT_3_ levels were measured using ECLIA. The following reference values were used: TSH = 0.465–4.68 Miu/mL, FT_3_ = 3.69–10.4 pmol/L, and FT_4_ = 10–28.2 pmol/L [31]. Thyroid ultrasonography was performed in all cases to measure the thyroid volume and study the changes in parenchymal density. The presence of an enlarged thyroid (thyroid gland volume > 97th age-related percentile) with diffuse hypo-echogenicity and/or diffuse micronodules confirmed the diagnosis of autoimmune thyroiditis.

The diagnosis of retinopathy was established after funduscopic examination conducted by a trained ophthalmologist who specialized in diabetic eye complications. Podiatric examination was performed according to the standardized protocol and included examinations of vibration and tactile, pain, and thermic sensations; photoplethysmography; and measurement of ankle and toe blood pressure to determine the ankle/brachial and toe/brachial indexes, respectively. Ewing’s battery test was performed to confirm the diagnosis of cardiac autonomic neuropathy. In some cases, echocardiography and Doppler ultrasound of the carotid artery were used to determine the severity of macrovascular complications.

### Assessment of sexual dysfunction

For assessment of sexual dysfunction, we used two internationally validated questionnaires: FSDS-R and FSFI [32, 33]. The original FSDS scale was created to measure distress, which is one of the most important symptoms of FSD [28]. We used the revised version (FSDS-R), comprising 13 questions, where the degree of distress is measured as a frequency. The response scale for each question’s item ranges between 0 (never) and 4 (always). The items are summed up to obtain a total score ranging from 0 to 52. The cut-off score is ≥ 11 [32].

The European version of the FSFI was translated, reviewed, and approved by the Research Committee of the Timisoara County Emergency Hospital for use with the Romanian population. The FSFI consists of 19 questions on six features of the female sexual response: desire, arousal, lubrication, orgasm, satisfaction, and pain. The answers are assessed according to how often each feature occurs. For the FSFI, the minimum score is 2 and the maximum is 36, with the cut-off score of 26.5 [33]. The scale includes questions on six domains, namely desire, subjective arousal, lubrication, orgasm, satisfaction, and pain. The individual domain scores are as follows: 1.2–6 for desire; 0–6 for arousal, lubrication, orgasm, and pain; and 0.8–6 for satisfaction. Low scores indicate high symptom burden [33]. The patients were asked to complete these questionnaires at home and return them after 4 weeks. Patients who did not return the questionnaire on time were sent a reminder via phone.

### Assessment of depression

For assessing the level of depression, we used the BDI-II scale [34], comprising 21 questions, with a minimum score of 0 and a maximum of 63, and a cut-off score of  ≥ 17 [34]. The patients were asked to complete this questionnaire at home and return it after 4 weeks. Patients who did not return the questionnaire on time were sent a reminder via phone.

There are three versions of the BDI—the original BDI, published in 1961 [35] and the revised versions published as BDI-1A and BDI-II in 1978 and 1996, respectively [34]. The BDI is widely used as an assessment tool by health care professionals and researchers in a variety of settings. In its current version, the BDI-II is designed for individuals aged ≥ 13 years and comprises items related to emotional symptoms of depression such as hopelessness, irritability, and guilt; cognitive symptoms; feelings of being punished; and physical symptoms such as fatigue, weight loss, and lack of interest in sexual activity. When the test is scored, a value from 0 to 3 is assigned to each answer and the total score is compared with a key to determine depression severity. The standard cut-off scores are as follows: 0–9: minimal depression, 10–18: mild depression, 19–29: moderate depression, 30–63: severe depression. High total scores indicate severe depressive symptoms.

### Statistical analysis

Statistical analysis was performed using the SPSS v.20 software (SPSS Inc., Chicago, IL, USA) and the R software packages (v.3) for statistical computing. Continuous data with Gaussian distribution are presented as mean (± standard deviation [SD]) values, whereas continuous variables without Gaussian distribution are presented as median (interquartile range) values. Nominal data are presented as absolute frequencies (percentages). The condition of normality of continuous variable distribution was tested using the Kolmogorov–Smirnov test, while the equality of variances was tested using Levene’s test.

The significance of the differences between the groups of women with FSD and without FSD was assessed using Student's *t*-test for Gaussian populations, while the Mann–Whitney *U* test was used for non-Gaussian populations. To assess the statistical significance between percentages, Pearson’s chi-squared test or Fisher’s exact test was used. The impact of one or more confounding factors on the presence of FSD was assessed with both univariate and multivariate logistic regression models, while the goodness-of-fit was calculated using Nagelkerke's *R*^2^ method. We assessed the linearity of the continuous variables with respect to the logit of the dependent variable by applying the Box–Tidwell procedure. A p-value < 0.05 was considered significant, with a confidence level of 0.95 for estimating intervals.

## Results

Our study sample included 104 women with both T1DM and CAT, and a mean age of 31.24 ± 6.86 years. The average duration of DM was 18.72 ± 9.42 years and of thyroid disease was 10.76 ± 7.52 years. The control group included 101 women with only T1DM, with similar age and DM duration as the study sample, i.e., 30.79 ± 5.69 years and 19.68 ± 10.66 years, respectively. The general characteristics of both groups are presented in Table 1.

**Table 1:** General characteristics of the studied samples

Parameters	Controls (women with T1DM) (*n* = *101*)	Women with T1DM and CAT (*n* = *104*)	*p*-value
Actual age (years)^a^	30.79 ± 5.69	31.24 ± 6.86	0.61
DM onset age (years)^a^	10.81 ± 7.59	12.52 ± 11.67	0.21
CAT onset age (years)^a^	NA	20.46 ± 9.23	NA
BMI (kg/m^2^)^a^	23.61 ± 2.69	23.32 ± 3.32	0.49
TC (mg %)^a^	156.37 ± 40.97	171.035 ± 53.73	0.029
HDLc (mg %)^a^	54.53 ± 8.31	51.66 ± 19.04	0.16
LDLc (mg %)^a^	73.11 ± 33.31	102.11 ± 34.68	< 0.001
TG (mg %)^a^	143.60 ± 80.94	86.28 ± 53.66	< 0.001
Hypercholesterolemia^b^	15 (14.85%)	29 (27.88%)	0.023
Depression^b^	19 (18.8%)	43 (41.3%)	< 0.001
DM duration (years)^a^	19.68 ± 10.66	18.72 ± 9.42	0.49
Fasting glycemia (mg %)^a^	162.10 ± 86.74	179.11 ± 85.69	0.159
HbA1c (%)^a^	8.14 ± 2.88	9.9 ± 3.39	< 0.001
Use of insulin pump^c^	10 (9.9%)	16 (15.4%)	0.238
Thyroid disease duration (years)^a^	NA	10.76 ± 7.52	NA
TSH (Miu/ml)^a^	2.17 ± 1.25	11.18 ± 18.21	< 0.001
FT_4_ (pmol/l)^a^	11.46 ± 5.35	9.62 ± 6.09	0.021
ANTITPO AB (UI/ml)^a^	9.29 ± 7.96	360.81 ± 345.94	< 0.001
ANTITG AB (UI/ml)^a^	25.12 ± 12.93	410.36 ± 410.45	< 0.001

*DM* diabetes mellitus, *CAT* chronic autoimmune thyroiditis, *BMI* body mass index, *TC* total cholesterol, *HDLc* high-density lipoprotein cholesterol, *LDLc* low-density lipoprotein cholesterol, *TG* triglycerides, *TSH* thyroid-stimulating hormone, *FT*_*4*_ thyroxine

^a^Continuous variables (with Gaussian distribution) are indicated as mean (± SD)

^b^Categorical variables are presented as percentage (absolute frequency) in the sample

The glycemic balance expressed by the average glycemic level and HbA1c was 179.11 ± 85.69 mg% and 9.9 ± 3.39%, respectively, with no significant differences between the groups. Microvascular complications were present in 24.03% of the patients, and macrovascular complications, in 15.38%. Diabetic polyneuropathy was present in 22.11% of the patients and retinopathy, in 15.38%. Further, 13.46% of the patients had chronic ischemic cardiac disease, while 4.30% had hypertension. In the control group, 6.9% of the patients had diabetic polyneuropathy, and 7.9% had retinopathy. Most of the patients were treated with intensified insulin therapy with four daily injections, while 15.4% used an insulin pump (Table 1). We determined thyroid function according to average TSH and FT_4_ levels, which were 11.18 ± 18.21 Miu/mL and 9.62 ± 6.09 pmol/L, respectively. We recorded the anti-TPO antibody and anti-Tg antibody levels as well as the type of treatment received by each patient (Table 1).

We observed that 49.04% of the T1DM and CAT patients presented with FSD. Precisely, the distribution of the FSD components was as follows: 56.86%, loss of libido; 47.05%, orgasm; 43.13%, lubrication; 33.33%, arousal; 19.6%, pain; and 47.05%, satisfaction problems. When we stratified the parameters of sexual function by the presence of CAT, we observed that significantly more patients with hypothyroidism than those with euthyroidism presented with FSD. Precisely, the FSFI (24.00 [22.80–30.00] vs. 28.80 [25.20–32.30]; Mann–Whitney *U* test, *U* = 821.00; p = 0.001) and the FSDS-R score were significantly higher in the former than in the latter (41.00 [8.00–43.00] vs. 8.00 [4.00–40.00]; Mann–Whitney *U* test, *U* = 1,929.00; p < 0.001). We observed that most of the parameters of FSD were similar between the control group patients and the patients with euthyroidism, e.g., there were no significant differences in the proportion of patients presenting with dysfunctions of desire, arousal, lubrication, satisfaction, and pain as well as in the FSDI (28.80 [26.60–30.00] vs. 28.80 [25.20–32.30]; Mann–Whitney *U* test, *U* = 2,875.00; p = 0.108) and FSDS-R scores (8.00 [6.00–10.00] vs. 8.00 [4.00–40.00], Mann–Whitney *U* test, *U* = 2,497.00; p = 0.928). The percentage of each component, along with the p-values from the comparisons between controls, patients with euthyroidism, and patients with hypothyroidism are presented in Table 2.

**Table 2:** Description of sexual function stratified by the presence of hypothyroidism

Parameters^a^	Controls (*n* = *101*)	Euthyroidism (*n* = *49*)	Hypothyroidism (*n* = *55*)	*p*-value^b^
FSFI score	28.80 (26.60–30.00)	28.80 (25.20–32.30)	24.00 (22.80–30.00)	< 0.001
Desire	9 (8.9%)	8 (16.3%)	21 (38.2%)	< 0.001
Arousal	7 (6.9%)	4 (8.2%)	14 (25.5%)	0.002
Lubrication	6 (5.9%)	3 (6.1%)	19 (34.5%)	0.001
Orgasm	8 (7.9%)	4 (8.2%)	20 (36.4%)	0.001
Satisfaction	10 (9.9%)	2 (4.1%)	19 (34.5%)	< 0.001
Pain	1 (1%)	1 (2.0%)	9 (16.4%)	< 0.001
FSDS-R score	8.00 (6.00–10.00)	8.00 (4.00–40.00)	41.00 (8.00–43.00)	< 0.001

*FSFI* Female Sexual Function Index, *FSDS-R* Female Sexual Distress Scale-Revised

^a^Continuous variables (with non-Gaussian distribution) are indicated as median (interquartile range) and categorical variables, as percentage (absolute frequency) in the sample

^b^p-value was computed by independent-samples Kruskal–Wallis test for continuous variables (with non-Gaussian distribution) and Pearson’s chi-squared (or Fisher’s exact) test for nominal variables

We compared the principal parameters described above between T1DM patients with FSD and those without FSD. Patients with FSD were significantly older than patients without FSD (33.00 [31.00–35.00] years vs. 28.00 [22.00–30.00] years; p < 0.001). In addition, the presence of FSD was associated with DM duration (24.00 [21.00–28.00] years vs. 20.00 [9.00–25.50] years; p = 0.005). On the contrary, we observed that the presence of FSD was not associated with high HbA1c, fasting glycemia, postprandial glycemia, and total cholesterol (HDLc, LDLc, TG) levels.

The parameters describing thyroid function were significantly different between patients with and those without FSD; precisely, TSH level was significantly higher in patients with FSD (7.00 [6.00–13.38] vs. 1.88 [1.11–3.08], p < 0.001), while the FT_4_ level was significantly lower (7.90 [1.20–12.80] vs. 12.50 [10.95–14.55], p < 0.001). The detailed comparison between patients with vs. without FSD can be found in Table 3.

**Table 3:** Comparative description of patients with FSD vs. without FSD

Parameters^a^	With FSD (*n* = *85*)	Without FSD (*n* = *120*)	p-value^b^
Age (years)	33.00 (31.00–35.00)	28.00 (22.00–30.00)	< 0.001
BMI (kg/m^2^)	23.77 (21.92–27.34)	22.49 (19.85–24.15)	0.020
TC (mg %)	167.00 (140.00–200.00)	161.50 (127.09–192.50)	0.362
HDLc (mg %)	55.50 (50.10–62.00)	54.75 (48.90–60.00)	0.550
LDLc (mg %)	94.80 (58.00–120.80)	84.30 (57.45–110.00)	0.275
TG (mg %)	97.00 (72.00–148.00)	100.00 (71.50–143.50)	0.824
DM duration (years)	26.00 (25.00–31.00)	20.00 (9.00–25.50)	0.005
Thyroid disease duration (years)	15.00 (14.00–18.00)	14.00 (11.00–16.00)	0.431
TSH (μ/ml)	7.00 (6.00–13.38)	1.88 (1.11–3.08)	< 0.001
FT_4_ (pmol/l)	7.90 (1.20–12.80)	12.50 (10.95–14.55)	< 0.001
ANTITPO AB (UI/ml)	360.00 (13.15–618.00)	319.00 (13.15–618.00)	0.552
ANTITG AB (UI/ml)	90.00 (32.00–816.00)	237.00 (39.45–923.45)	0.313
Fasting glycemia (mg %)	162.00 (127.00–244.00)	160.00 (120.00–200.00)	0.409
Postprandial glycemia (mg %)	147.42 (119.00–198.90)	141.11 (102.00–172.00)	0.408
HbA1c (%)	9.20 (7.95–13.55)	8.75 (7.40–11.15)	0.149
Diabetes microvascular complications			
Retinopathy	15 (17.6%)	9 (7.5%)	0.026
Nephropathy	1 (1.17%)	1 (0.83%)	0.978
Diabetic polyneuropathy	18 (21.2%)	12 (10%)	0.026
Diabetes macrovascular complications	11 (12.94%)	5 (4.16%)	0.086
Hypertension	18 (21.17%)	10 (8.33%)	0.030
Chronic cardiac ischemic disease	10 (11.76%)	4 (3.33%)	0.089
Hypertension treatment (beta-blockers)	11 (12.94%)	5 (9.4%)	0.086
Use of insulin pump	21 (24.7%)	5 (4.2%)	< 0.001
Goiter	21 (24.7%)	10 (8.33%)	0.018
Depression	57 (67.05%)	5 (4.2%)	< 0.001

*DM* diabetes mellitus, *FSD* female sexual dysfunction, *BMI* body mass index, *TC* total cholesterol, *HDLc* high-density lipoprotein cholesterol, *LDLc* low-density lipoprotein cholesterol, *TG* triglycerides, *TSH* thyroid-stimulating hormone, *FT*_*4*_ thyroxine

^a^Continuous variables (with non-Gaussian distribution) are indicated as median (interquartile range) and categorical variables, as percentage (absolute frequency) in the sample

^b^p-value was computed by Mann–Whitney *U* test for continuous variables (with non-Gaussian distribution) and Pearson’s chi-squared (or Fisher's exact) test for nominal variables

A significantly higher number of patients with FSD than those without FSD presented with depressive disorders (67.05 vs. 4.2%, p < 0.001). Moreover, a significantly higher number of patients with FSD and CAT than those without CAT presented with goiter (24.7 vs. 8.33%, p = 0.018). Interestingly, a significantly higher number of patients using insulin pumps than those using injections presented with FSD (24.7 vs. 4.2%, p < 0.001).

### Univariate logistic regressions

We used univariate logistic regression models to examine the impact of patients’ characteristics on the presence of FSD. We observed that age was a significant risk factor for FSD (OR, 1.077; 95% CI, 1.027–1.129; p = 0.002). DM duration (OR, 1.030; 95% CI, 1.001–1.059; p = 0.026) and high BMI were also significant risk factors for FSD (OR, 1.121; 95% CI, 1.011–1.241; p = 0.022).

Regarding DM management and associated complications, we observed that HbA1c levels were not a significant risk factor for FSD (OR, 1.056; 95% CI, 0.970–1.149; p = 0.209). On the contrary, diabetic polyneuropathy was a significant risk factor for FSD (OR, 2.418; 95% CI, 1.096–5.336; p = 0.029).

High level of TSH was a significant risk factor for FSD (OR, 1.085; 95% CI, 1.030–1.143; p = 0.002), while high level of FT_4_ was a significant protective factor against the risk of developing FSD (OR, 0.889; 95% CI, 0.827–0.956; p = 0.002). Moreover, the presence of goiter was a significant risk factor for FSD (OR, 3.010; 95% CI, 1.241–7.298; p = 0.015).

Patients with depressive disorder had 30.412 times higher odds of developing FSD than patients without depression (95% CI, 9.301–95.126). Moreover, patients using insulin pumps had 7.547 times higher odds of developing FSD than patients who did not (95% CI, 2.716–20.973).

### Multivariate logistic regression

We performed multivariate logistic regression analysis to assess the effects of patient characteristics on the presence of FSD. We applied the Bonferroni correction resulting in statistical significance being accepted when p < 0.01. Thus, all continuous independent variables were found to be linearly related to the logit of the dependent variable.

The obtained model was statistically significant (χ^2^(9) = 96.581, p < 0.001). Nagelkerke’s R^2^ coefficient indicated that predictor variables explained 80.7% of the variance in the presence of FSD. The statistically significant predictor variables were age, DM duration, CAT, diabetic polyneuropathy, depression, and use of insulin pump (Table 4).

**Table 4:** Predictors of the presence of FSD in women with T1DM (multivariate logistic regression model; Nagelkerke’s R^2^ = 0.807)

Predictor variable	Crude OR	95% CI		Adjusted OR	95% CI	
		Lower	Upper		Lower	Upper
Age (years)*	1.077	1.027	1.129	1.162	1.012	1.455
DM duration (years)*	1.030	1.001	1.059	1.197	1.060	1.351
BMI*	1.121	1.011	1.241	1.248	1.120	1.501
Fasting glycemia (mg/dL)	1.000	0.997	1.003	1.012	0.712	1.027
HbA1c (%)	1.056	0.970	1.149	1.118	0.703	1.779
Diabetic polyneuropathy*	2.418	1.096	5.336	2.543	1.854	4.231
CAT*	1.887	1.341	2.655	2.954	1.631	3.885
Goiter	3.010	1.241	7.298	0.309	0.044	2.170
Depression*	30.412	9.301	95.126	3.463	2.072	3.945
Use of insulin pump*	7.547	2.716	20.973	2.431	1.705	3.132

*Predictor variable is significant both independently and as a co-factor

*DM* diabetes mellitus, *BMI* body mass index, *CAT* chronic autoimmune thyroiditis

Older women with T1DM had 1.162 times higher odds of developing FSD than younger women with T1DM (95% CI, 1.012–1.455; p = 0.002). Moreover, women with long DM duration had 1.197 times higher odds of developing FSD than women with short DM duration (95% CI, 1.060–1.351; p = 0.004). High BMI values predicted an increased likelihood of FSD (OR, 1.248; 95% CI, 1.120–1.501; p = 0.040).

Well-managed DM, as per HbA1c values, was not a risk factor for FSD (OR, 1.012; 95% CI, 0.712–1.027; p = 0.812). However, coexisting CAT was a significant risk factor for FSD (OR, 2.954; 95% CI, 1.631–3.885; p = 0.001). The women with goiter were not at a significantly high risk of developing FSD.

**Fig. 1:**
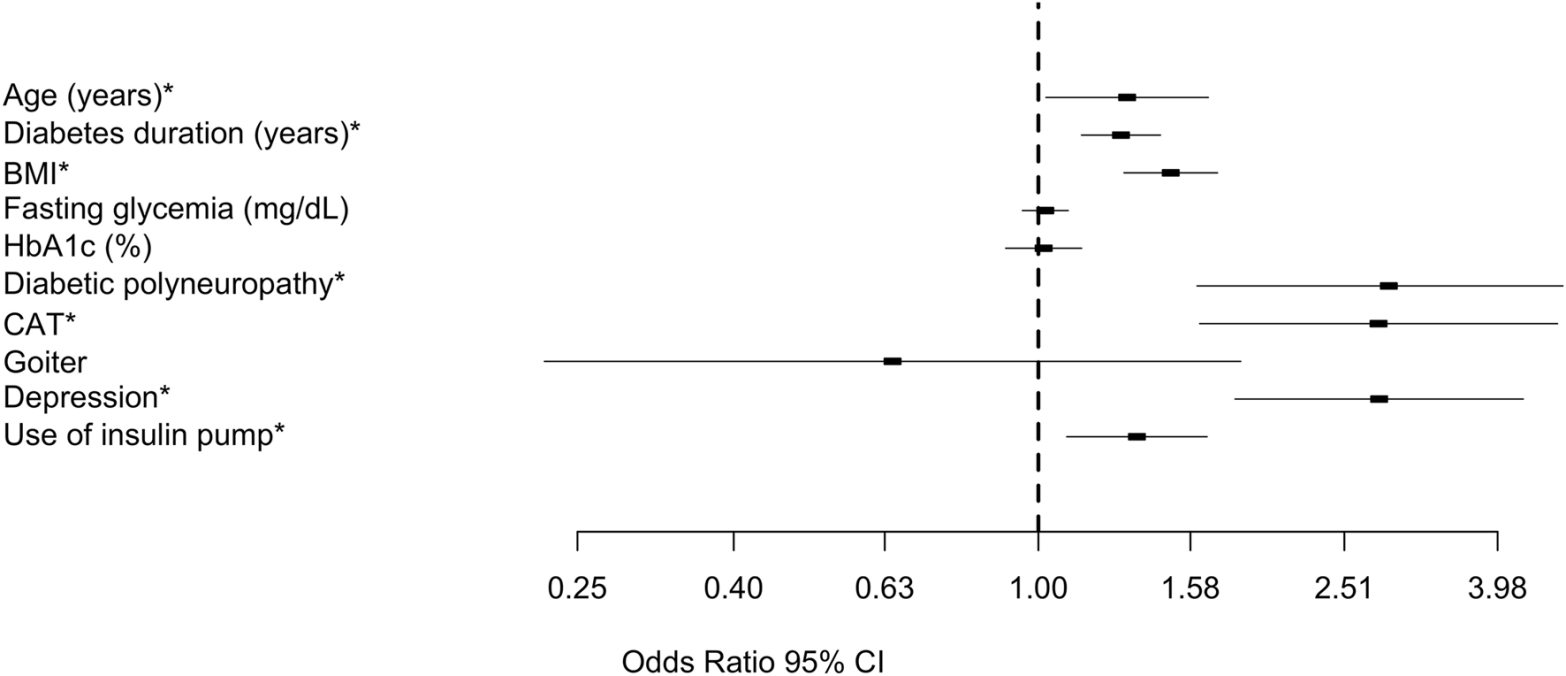
Risk analysis for FSD in T1DM and CAT patients. The risk is expressed per 1 percentage point increase in HbA1c and 1 mg/dL point increase in fasting glycemia. For polyneuropathy, CAT, goiter, depression and use of insulin pump, the risk is expressed as a dichotomous variable. *Predictor variable is significant both independently and as a co-factor. Abbreviations: BMI, body mass index; CAT, chronic autoimmune thyroiditis

Women with the DM complication of polyneuropathy had 2.543 times higher odds of developing FSD (95% CI, 1.854–4.231; p = 0.003). Depression was a significant risk factor for FSD (OR, 3.463; 95% CI, 2.072–3.945; p < 0.001). Moreover, women using an insulin pump had 2.431 times higher odds of developing FSD than patients not using it (95% CI, 1.705–3.132; p < 0.001) (Fig. 1).

### Discussion

In this study, we focused on sexual dysfunction in Romanian women with T1DM and CAT. We observed that a significantly higher number of patients with T1DM and CAT (49%) than those with T1DM only (33.7%; p = 0.025) presented with FSD. A significantly higher number of women with FSD in the group with T1DM and CAT than in the group with only T1DM complained of sexual problems: 27.9% vs. 8.9 (p < 0.001), desire; 23.1 vs. 7.9% (p = 0.003), orgasm; 21.2 vs. 5.9% (p = 0.002), lubrication; 17.3 vs. 6.9% (p = 0.023), arousal; 9.6 vs. 1% (p = 0.006), pain; and 20.2 vs. 9.9% (p = 0.040), satisfaction problems.

In Romania, there are no data on the prevalence and risk factors for FSD. The prevalence and type of sexual dysfunction vary worldwide. A difference was observed in the prevalence of FSD between healthy women and women with diabetes and/or thyroid diseases. According to recent studies, the prevalence of FSD is higher in patients with DM than in the general population. In women with T1DM, the prevalence of FSD varies between 18 and 71%, and the odds ratio is 2.27 in patients with FSD [36]. A study performed by Ezlin et al. reported that sexual dysfunction was more frequent in women with T1DM than in healthy women (27 vs. 15%), with a predominance of decreased lubrication [37]. In the Diabetes Control and Complications Trial (DCCT)/EDIC study, FSD was reported in 35% women, featuring loss of libido, orgasm disorders, impairment of lubrication and arousal, and pain during sexual activity [38]. Dimitropoulos et al. found that FSD characterized by altered desire, arousal, and satisfaction was present in 15.9% of the women with T1DM as opposed to in 2.1% of the women in the control group [39].

The prevalence of FSD was 46.1% in patients with thyroid as opposed to 20.7% in healthy patients [40]. In our study, the prevalence of FSD was higher because of the association between the two autoimmune diseases: T1DM and CAT. Both diseases can lead to atherosclerosis, one of the causes of FSD. The association between thyroid disease and vaginal lubrication and orgasm explains the high prevalence of these two disorders in our study. In women with T1DM, the main factors involved in FSD development are atherosclerotic process, nervous system impairment, and endothelial dysfunction [41]. Chronic hyperglycemia causes a decrease in mucus production and lubrication, resulting in an increased prevalence of bacterial and fungal infections. Owing to the atherosclerotic process, arterial blood flow is decreased in the female genital organs, causing a decrease in vaginal lubrication and sexual stimulation [41]. Moreover, hormonal imbalances often occur because of T1DM, which can induce a decline in sexual desire, inadequate lubrication of the vagina, orgasm disturbances, and coital pain. Besides the somatic causes, various psychosocial factors such as poor body self-perception, fear of hypoglycemia, feeling of guilt and disgrace, and discontentment can also cause FSD. Depression frequently accompanies diabetes and is associated with a lack of energy, absence of sexual desire, lack of self-esteem, difficulties in pleasure perception, and psychosocial problems causing irritability, which are risk factors for FSD [24]. Some drugs are also involved in the occurrence of FSD, namely barbiturates, benzodiazepines, tricyclic antidepressants, anti-lipid medications, beta-blockers, clonidine, digoxin, spironolactone, oral contraceptives, histamine H2–receptor blockers, indomethacin, ketoconazole, etc. In our study, the patients were treated with beta-blockers for hypertension, but we did not find any association between FSD and these medications. Another cause of the high prevalence of FSD in women with T1DM was the presence of chronic diabetic complications (neuropathy, in particular). We found an association between FSD and the presence of diabetic polyneuropathy as previously reported [42]. Neuropathy may decrease genital blood flow, leading to an impaired genital arousal response. Bock et al. studied FSD in the context of vaginal blood flow, clitoral sensitivity, and association with diabetes complications. They found that T1DM patients with retinopathy presented with a low vaginal pulse amplitude, while T1DM patients with neuropathy had a high sensation threshold for vibrotactile stimulation of the clitoris [43].

Another predictive factor of FSD was old age, given the hormonal changes with increasing age. In our study, this was a risk factor for FSD, similar to that previously reported [44]. Some previous studies explored the correlation between FSD and BMI. Among these, some showed no correlation between the two, while others found a correlation between FSFI with BMI, but not with the waist-hip ratio. From the six FSD parameters evaluated, four correlated with BMI (arousal, lubrication, orgasm, and sexual pleasure) [45]. We found a correlation between FSD and high BMI.

To evaluate the risk factors for FSD in women with T1DM, we also studied the DM duration**,** glycemic balance expressed by glycated hemoglobin level, and lipid profile. Over the last few years, T1DM treatment has been intensified with the introduction of insulin analogs, insulin pump therapy, and continuous glucose monitoring; hence, DM control has improved, resulting in a reduced impact on sexual life. Furthermore, the treatment of dyslipidemia has been intensified, and the targets for lipid parameters have been reduced, resulting in improved control of dyslipidemia. We found an association between DM duration and FSD, but not between glycated hemoglobin, lipid profile, and FSD. Moreover, previous studies on T1DM did not show a correlation between glycated hemoglobin and FSD [46]. Using an insulin pump was one of the risk factors for FSD in our study. Insulin pumps are perceived negatively with respect to sexual activity among women; further research is required regarding pump disconnection during sex.

Some studies reported an increase in FSD in women with T1DM and depression [46]. In our study, depression was a significant risk factor of FSD. Similarly, depression was the main risk factor for FSD in the study performed by Ezlin et al. [37], the DCCT/EDIC study [38], and the study performed by Dimitropoulos et al. [39]. Patients with T1DM experience a decrease in self-esteem and increased feelings of loneliness, isolation, or suffering as friends, family, or partners do not understand the effect of insulin administration on their lifestyle; the patients also experience loss of attractiveness due to weight gain secondary to insulin treatment.

In the univariate logistic regressions, we found that the presence of goiter was a significant risk factor for FSD, but it was not a predictive factor, as per the multivariate logistic regression. Other studies also showed that women with thyroid nodular goiter presented with a high risk of developing FSD [47].

T1DM is an autoimmune disease that can lead to other autoimmune diseases, usually CAT that develops to hypothyroidism over time. Several studies showed an influence of thyroid diseases on fertility (ovarian and menstruation cycles). In our study, coexisting CAT was a significant risk factor for FSD. Veronelli et al. observed that women with DM and hypothyroidism had low levels of arousal and desire. Furthermore, they also had depressive disorders. Thus, these patients were susceptible to the development of atherosclerosis [48].

### Limitations of the study

The first limitation of the current study is the number of participants. Intimate life is a very sensitive subject, and many women avoided responding to the questions about their sexual life, even under the condition of anonymity. Second, all participants were Caucasian; further studies are required with other ethnic groups (black race, Hispanic women, etc.).

Other limitations of our study include the administration of validated questionnaires for FSD assessment, which is a subjective assessment; in gynecological practice, there exist objective methods for FSD assessment, such as clitoral color, flow Doppler ultrasonography, and pulsatility index. Moreover, we did not investigate the impact of other psychological problems, personality types, and coping mechanisms on FSD.

## Conclusions

We found that women with T1DM and coexisting CAT were at a high risk of developing FSD. Therefore, they should be aware of the risk factors. Reducing exposure to depression-generating factors and exercising caution while using insulin pumps are important measures. Moreover, even if DM management is not a direct risk factor for FSD, DM complications, such as polyneuropathy, are significant risk factors; therefore, it is crucial to ensure glycemic control and optimal DM management.

## Data Availability

The data from this research study are publicly available in supporting files.

## Abbreviations

FSD: Female sexual dysfunction
DM: Diabetes mellitus
T1DM: Type 1 diabetes mellitus
CAT: Chronic autoimmune thyroiditis
OR: Odds ratio
CI: Confidence interval
BMI: Body mass index
FSFI: Female sexual function index
FSDS-R: Female sexual distress scale-revised
BDI-II: Beck's depression inventory-II scale
HDLc: High-density lipoprotein cholesterol
LDLc: Low-density lipoprotein cholesterol
TG: Triglycerides
TSH: Thyroid-stimulating hormone
FT_4_: Thyroxine

## References

[1] Nolen-Hoeksema S (2014). Abnormal psychology.

[2] Starc A, Jukić T, Poljšak B, Dahmane R (2018). Female sexual function and dysfunction: a cross-national prevalence study in Slovenia. Acta Clin Croat.

[3] Tafazoli M, Parnan A, Azmoude E (2017). Sexual function and quality of life in diabetic women referring to health care centers in Mashhad. J Educ Health Promot.

[4] Popovic DS, Majic A, Prodanovic-Simeunovic J (2019). The sexual dysfunction in females with type 1 diabetes: still an underestimated issue?. Diabetes Updates.

[5] DeFronzo RA, Ferrannini E, Zimmet P (2015). International textbook of diabetes mellitus, 2 volume set.

[6] Holt RIG, Cockram C, Flyvbjerg A (2017). Textbook of diabetes.

[7] Holman N, Young B, Gadsby R (2015). Current prevalence of Type 1 and Type 2 diabetes in adults and children in the UK. Diabet Med J Br Diabet Assoc.

[8] Krzewska A, Ben-Skowronek I (2016). Effect of Associated Autoimmune Diseases on Type 1 Diabetes Mellitus Incidence and Metabolic Control in Children and Adolescents. Biomed Res Int.

[9] Denzer C, Karges B, Nake A (2013). Subclinical hypothyroidism and dyslipidemia in children and adolescents with type 1 diabetes mellitus. Eur J Endocrinol.

[10] International Diabetes Federation (2017). IDF Diabetes Atlas.

[11] Moţa E, Popa SG, Moţa M (2015). Prevalence of chronic kidney disease and its association with cardiometabolic risk factors in the adult Romanian population: the PREDATORR study. Int Urol Nephrol.

[12] Rahmanian E, Salari N, Mohammadi M (2019). Evaluation of sexual dysfunction and female sexual dysfunction indicators in women with type 2 diabetes: a systematic review and meta-analysis. Diabetol Metab Syndr..

[13] Amidu N, Owiredu WK, Alidu H (2013). Association between metabolic syndrome and sexual dysfunction among men with clinically diagnosed diabetes. Diabetol Metab Syndr.

[14] Meeking DR, Fosbury JA, Cummings MH (2013). Sexual dysfunction and sexual health concerns in women with diabetes- review. Practical Diabetes.

[15] Hotaling JM, Sarma AV, Patel DP (2016). Cardiovascular autonomic neuropathy, sexual dysfunction, and urinary incontinence in women with type 1 diabetes. Diabetes Care.

[16] Maseroli E, Scavello I, Vignozzi L (2018). Cardiometabolic risk and female sexuality-Part I. Risk factors and potential pathophysiological underpinnings for female vasculogenic sexual dysfunction syndromes. Sex Med Rev..

[17] McCool-Myers M, Theurich M, Zuelke A (2018). Predictors of female sexual dysfunction: a systematic review and qualitative analysis through gender inequality paradigms. BMC Women's Health.

[18] Brotto L, Atallah S, Johnson-Agbakwu C (2016). Psychological and interpersonal dimensions of sexual function and dysfunction. Int Consult Sexual Med Rep.

[19] Bhasin S, Enzlin P, Coviello A, Basson R (2007). Sexual dysfunction in men and women with endocrine disorders. Lancet.

[20] Carani C, Isidori AM, Granata A (2005). Multicenter study on the prevalence of sexual symptoms in male hypo- and hyperthyroid patients. J Clin Endocrinol Metab.

[21] Atis G, Dalkilinc A, Altuntas Y (2011). Hyperthyroidism: a risk factor for female sexual dysfunction. J Sex Med.

[22] Atis G, Dalkilinc A, Altuntas Y (2010). Sexual dysfunction in women with clinical hypothyroidism and subclinical hypothyroidism. J Sex Med.

[23] Gabrielson AT, Sartor RA, Hellstrom WJG (2019). The Impact of Thyroid Disease on Sexual Dysfunction in Men and Women. Sex Med Rev.

[24] Pontiroli AE, Cortelazzi D, Morabito A (2013). Female sexual dysfunction and diabetes: A systematic review and metaanalysis. J Sex Med.

[25] Zamponi V, Mazzilli R, Bitterman O (2020). Association between type 1 diabetes and female sexual dysfunction. BMC Women's Health.

[26] Mollaoğlu M, Tuncay FÖ, Fertelli TK (2013). Investigating the sexual function and its associated factors in women with chronic illnesses. J Clin Nurs.

[27] Neufeld M, Maclaren N, Blizzard R (1980). Autoimmune polyglandular syndromes. Pediatr Ann.

[28] Eden KJ, Wylie KR (2009). Quality of sexual life and menopause. Women's Health.

[29] Kingsberg SA (2002). The impact of aging on sexual function in women and their partners. Arch Sex Behav.

[30] American Diabetes Association (2019). Classification and diagnosis of diabetes: standards of medical care in diabetes. Diabetes Care.

[31] American Thyroid Association (2019). Thyroid function tests.

[32] Derogatis LR, Rosen R, Leiblum S (2002). The Female Sexual Distress Scale (FSDS): Initial validation of a standardized scale for assessment of sexually related personal distress in women. J Sex Marital Ther.

[33] Rosen R, Brown C, Heiman J (2000). The Female Sexual Function Index (FSFI): A multidimensional self-report instrument for the assessment of female sexual function. J Sex Marital Ther.

[34] Beck AT, Ward CH, Mendelson M (1961). An inventory for measuring depression. Arch Gen Psychiatr.

[35] Beck AT, Steer RA, Ball R (1996). Comparison of Beck Depression Inventories e I A and e II in psychiatric outpatients. J Pers Assess.

[36] Stechova K, Mastikova L, Urbaniec K (2019). Sexual dysfunction in women treated for type 1 diabetes and the impact of coexisting thyroid disease. Sex Med..

[37] Enzlin P, Mathieu C, Van Den Bruel A, Vanderschueren D, Demyttenaere K (2003). Prevalence and predictors of sexual dysfunction in patients with type 1 diabetes. Diabetes Care.

[38] Enzlin P, Rosen R, Wiegel M, Brown J, Wessells H (2009). Sexual dysfunction in women with type 1 diabetes. Diabetes Care.

[39] Dimitropoulos K, Bargiota A, Mouzas O, Melekos M, Tzortzis V (2012). Sexual functioning and distress among premenopausal women with uncomplicated type 1 diabetes. J Sex Med.

[40] Pasquali D, Maiorino MI, Renzullo A, Bellastella G, Accardo G, Esposito D (2013). Female sexual dysfunction in women with thyroid disorders. J Endocrinol Invest.

[41] Clayton AH (2010). The pathophysiology of hypoactive sexual desire disorder in 34 women. Int J Gynaecol Obstet.

[42] Bak E, Marcisz C, Krzeminska S, Dobrzyn-Matusiak D, Foltyn A (2018). Does type 1 diabetes modify sexuality and mood of women and men?. Int J Environ Res Public Health.

[43] Both S, Ter Kuile M, Enzlin P, Dekkers O, van Dijk M (2015). Sexual response in women with type 1 diabetes mellitus: a controlled laboratory study measuring vaginal blood flow and subjective sexual arousal. Arch Sex Behav.

[44] Mitchell KR, Mercer CH, Ploubidis GB (2013). Sexual function in Britain: findings from the third National Survey of Sexual Attitudes and Lifestyles (Natsal-3). Lancet.

[45] Maiorino MI, Bellastella G, Castaldo F, Petrizzo M, Giugliano D (2017). Sexual function in young women with type 1 diabetes: the METRO study. J Endocrinol Invest.

[46] Tagliabue M, Gottero C, Zuffranieri M (2011). Sexual function in women with type 1 diabetes matched with a control group: depressive and psychosocial aspects. J Sex Med.

[47] Bates JN, Kohn TP, Pastuszak AW (2020). Effect of thyroid hormone derangements on sexual function in men and women. Sexual Medicine Reviews.

[48] Veronelli A, Mauri C, Zecchini B (2009). Sexual dysfunction is frequent in premenopausal women with diabetes, obesity, and hypothyroidism, and correlates with markers of increased cardiovascular risk. A preliminary report J Sex Med.

